# Genome-Wide Profiling Identified a Set of miRNAs that Are Differentially Expressed in Glioblastoma Stem Cells and Normal Neural Stem Cells

**DOI:** 10.1371/journal.pone.0036248

**Published:** 2012-04-30

**Authors:** Ming-Fei Lang, Su Yang, Chunnian Zhao, Guoqiang Sun, Kiyohito Murai, Xiwei Wu, Jinhui Wang, Hanlin Gao, Christine E. Brown, Xiaoxuan Liu, Jiehua Zhou, Ling Peng, John J. Rossi, Yanhong Shi

**Affiliations:** 1 Department of Neurosciences, Center for Gene Expression and Drug Discovery, Cancer Center, Beckman Research Institute of City of Hope, Duarte, California, United States of America; 2 Irell & Manella Graduate School of Biological Sciences, Beckman Research Institute of City of Hope, Duarte, California, United States of America; 3 Department of Molecular Medicine, Beckman Research Institute of City of Hope, Duarte, California, United States of America; 4 DNA sequencing/Solexa Core, Beckman Research Institute of City of Hope, Duarte, California, United States of America; 5 Department of Hematology and Hematopoietic Cell Transplantation, Beckman Research Institute of City of Hope, Duarte, California, United States of America; 6 Centre Interdisciplinaire de Nanoscience de Marseille, CNRS UMR 7325, Aix-Marseille University, Marseille, France; 7 Department of Molecular and Cellular Biology, Beckman Research Institute of City of Hope, Duarte, California, United States of America; H.Lee Moffitt Cancer Center & Research Institute, United States of America

## Abstract

A major challenge in cancer research field is to define molecular features that distinguish cancer stem cells from normal stem cells. In this study, we compared microRNA (miRNA) expression profiles in human glioblastoma stem cells and normal neural stem cells using combined microarray and deep sequencing analyses. These studies allowed us to identify a set of 10 miRNAs that are considerably up-regulated or down-regulated in glioblastoma stem cells. Among them, 5 miRNAs were further confirmed to have altered expression in three independent lines of glioblastoma stem cells by real-time RT-PCR analysis. Moreover, two of the miRNAs with increased expression in glioblastoma stem cells also exhibited elevated expression in glioblastoma patient tissues examined, while two miRNAs with decreased expression in glioblastoma stem cells displayed reduced expression in tumor tissues. Furthermore, we identified two oncogenes, NRAS and PIM3, as downstream targets of miR-124, one of the down-regulated miRNAs; and a tumor suppressor, CSMD1, as a downstream target of miR-10a and miR-10b, two of the up-regulated miRNAs. In summary, this study led to the identification of a set of miRNAs that are differentially expressed in glioblastoma stem cells and normal neural stem cells. Characterizing the role of these miRNAs in glioblastoma stem cells may lead to the development of miRNA-based therapies that specifically target tumor stem cells, but spare normal stem cells.

## Introduction

According to the World Health Organization (WHO) classification of tumors, a grading scheme, which represents a malignancy scale and a key factor influencing the choice of therapies, has been successfully applied to astrocytomas, the most common type of glioma [1]. The WHO defines pilocytic astrocytoma as grade I, diffuse astrocytoma as grade II, anaplastic astrocytoma as grade III, and glioblastoma as grade IV, the most malignant grade [1]. Glioblastoma is the most common and aggressive primary brain tumor with median survival time of 14 months after diagnosis [1]. Until now, no effective treatment has been developed for glioblastoma patients. The goal of our research is to identify novel molecular targets for this malignant tumor, and thus glioblastoma is the main interest of this study. Recent studies have led to the hypothesis that glioblastomas are maintained by a small population of cancer stem cells that retain stem cell properties, are highly tumorigenic, and display increased resistance to radiation and chemotherapy [2]–[4]. These treatment-resistant tumor cell subpopulations are the cell populations that effective therapies must target [4].

miRNAs are short 20–22 nucleotide RNA molecules that are expressed in a tissue-specific and developmentally-regulated manner and function as negative regulators of gene expression in a variety of eukaryotes. miRNAs are involved in numerous cellular processes including development, proliferation, and differentiation [5], [6], [7]. Increasing evidence has linked miRNAs to cancer [8]. miRNAs are important regulators of many key pathways implicated in tumor pathogenesis [9]. They can function as either oncogenes or tumor suppressors in various tumors [10].

Recently, miRNAs have been shown to be differentially expressed in glioblastoma tissues compared to normal brain tissues. For example, miRNA 21 is overexpressed in glioblastoma tissues, relative to surrounding normal brain tissues [11]. miR-26a is also amplified in glioblastoma tissues. By targeting the tumor suppressor Pten, overexpression of miR-26a facilitates tumorigenesis and predicts a poor survival [12], [13]. On the other hand, miR-124, miR-137 and miR-451 exhibit reduced expression in malignant glioblastoma tissues relative to normal brain tissues [14], [15]. The expression of these miRNAs is also reduced in glioblastoma stem cells relative to bulk tumor cells. Overexpression of these miRNAs in glioblastoma stem cells inhibits cell proliferation and induces neural differentiation, suggesting a tumor suppressor role for these miRNAs. These studies suggest that some miRNAs may be used as therapeutic agents for targeting glioblastoma stem cells. However, brain tumor stem cells share a core developmental program with normal neural stem cells [10]. Optimal therapies should target tumor stem cells, but spare normal stem cells. Therefore, identifying miRNAs that are differentially expressed in glioblastoma stem cells and normal neural stem cells becomes essential for the development of optimal miRNA-based therapies for glioblastoma patients.

In this study, we present the results of a genome-wide miRNA expression profiling in human glioblastoma stem cells and normal neural stem cells using combined miRNA microarray and deep sequencing analyses. This study led to the identification of eight miRNAs that are substantially up-regulated and two miRNAs that are significantly down-regulated in glioblastoma stem cells, relative to normal neural stem cells. Differential expression of four of these miRNAs, 2 up-regulated and 2 down-regulated, was further validated by real-time RT-PCR in both glioblastoma stem cells and glioblastoma patient tumor tissues. Moreover, we demonstrate that these up-regulated or down-regulated miRNAs inhibit the expression of genes that are involved in tumor suppression or tumorigenesis, respectively.

## Results

### Differential miRNA expression in glioblastoma stem cells and normal neural stem cells

**Figure 1:**
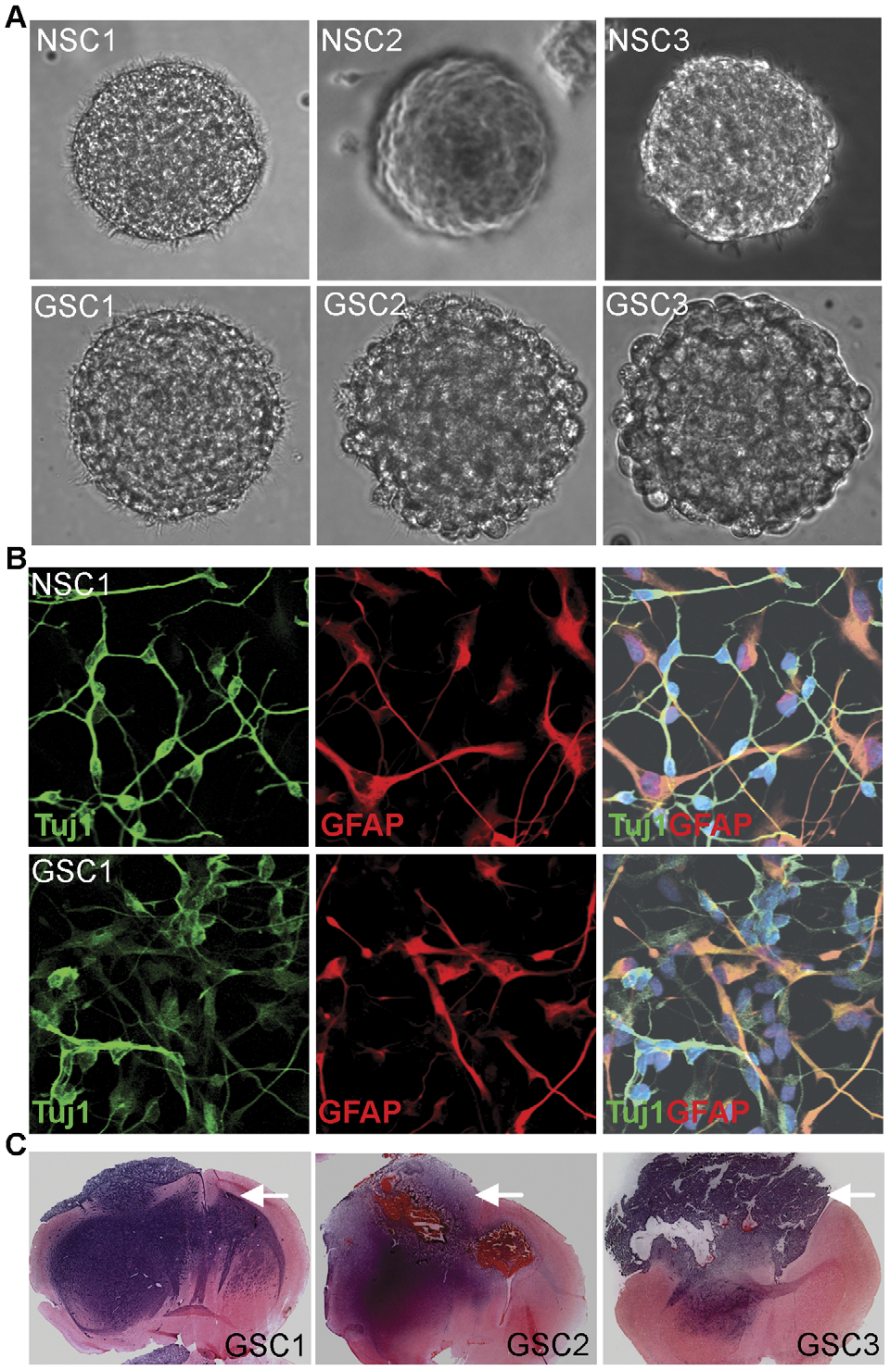
The morphology, differentiation and growth curve of glioblastoma stem cells (GSCs) and neural stem cells (NSCs). **A.** Representative images of neurospheres from normal human neural stem cell lines 1–3 (NSC1–3) and glioblastoma stem cell lines 1–3 (GSC1–3). **B.** The multipotency of NSCs and GSCs. When induced into differentiation, both NSCs and GSCs gave rise to Tuj1+ neurons (green) and GFAP+ astrocytes (red). Representative images of NSC1 and GSC1 differentiation were shown. Nuclear Dapi staining was shown in blue. **C.** H&E staining of coronal sections from GSC-transplanted brains. The tumor region was indicated by an arrow, shown in dark purple color.

In order to identify miRNAs that are differentially expressed in glioblastoma stem cells and normal neural stem cells, we established three primary glioblastoma stem cell lines and three normal human neural stem cell lines to determine if there were significant differences in miRNA expression in tumor stem cells from normal stem cells. Human primary glioblastoma stem cells were derived from newly diagnosed glioblastoma multiforme IV patients and cultured in DMEM/F12 media supplemented with epithelial growth factor (EGF), fibroblast growth factor (FGF), and B27 supplement. Human normal neural stem cells were derived from normal human brain tissues and cultured in the same media. Both glioblastoma stem cells and normal neural stem cells grew as neurospheres under the culture condition (Fig. 1A). Both types of cells are multipotent, having the ability to differentiate into Tuj1-positive neurons and GFAP-positive astrocytes when induced into differentiation using fetal bovine serum and all-trans retinoic acid (Fig. 1B). However, the glioblastoma stem cells were able to generate tumors when transplanted to the immunodeficient NSG mice (Fig. 1C), whereas the human neural stem cells did not (data not shown).

Combined microarray and deep sequencing analyses were performed to determine the expression profile of miRNAs in glioblastoma stem cells and normal neural stem cells. Total RNAs were prepared from both glioblastoma stem cells and neural stem cells for miRNA microarray analysis. In microarray analysis, we identified 10 miRNAs that are more than 5-fold up-regulated in glioblastoma stem cells and 8 miRNAs that are more than 5-fold down-regulated in glioblastoma stem cells, relative to neural stem cells (Table 1). The differentially expressed miRNAs that exhibit more than 1.5-fold difference in the expression between glioblastoma stem cells and neural stem cells were shown in Table S1.

**Table 1:** Up-regulated and down-regulated miRNAs in human glioblastoma stem cells, compared to human neural stem cells.

miRNA	Chromosomal location	Fold-Change	*p*-value
up-regulated			
hsa-miR-10a	17q21.32	93.65	3.28E-10
hsa-miR-10b	2q31.1	90.38	2.06E-09
hsa-miR-140-3p	16q22.1	14.10	1.62E-10
hsa-miR-140-5p	16q22.1	12.19	5.56E-09
hsa-miR-204	9q21.12	9.05	4.08E-08
hsa-miR-424	Xq26.3	8.38	2.53E-08
hsa-miR-455-5p	9q32	5.87	1.48E-05
hsa-miR-455-3p	9q32	5.41	9.32E-05
down-regulated			
hsa-miR-371-5p	19q13.42	−15.27	8.52E-11
hsa-miR-124-1*	8p23.1	−13.37	8.33E-05
hsa-miR-124-2*	8q12.3		
hsa-miR-124-3*	20q13.33		
hsa-miR-335	7q32.2	−13.24	1.43E-09
hsa-miR-492	12q22	−7.77	5.38E-08
hsa-miR-874	5q31.2	−6.76	1.53E-08
hsa-miR-30b*	8q24.22	−6.54	2.73E-09
hsa-miR-602	9q34.3	−5.75	2.63E-08

* hsa-miR-124 is transcribed from three chromosomal locations, but the mature sequences are the same.

Using an Illumina Genome Analyzer II (GAII) sequencing system, we performed the whole-genome small RNA sequencing in glioblastoma stem cells and neural stem cells. Significantly more miRNAs were detected to be differentially expressed in glioblastoma stem cells and neural stem cells in deep sequencing analysis. For example, deep sequencing analysis revealed 105 miRNAs that were up-regulated more than 5-fold in glioblastoma stem cells. However, microarray analysis revealed only 10 miRNAs showing more than 5-fold increase in glioblastoma stem cells. Interestingly, 8 out of the 10 miRNAs that were up-regulated more than 5-fold in microarray analysis also exhibited significantly increased expression in deep sequencing analysis (Table 2). Two of the miRNAs that had more than 5-fold decrease of expression in microarray analysis also showed more than 5-fold reduction of expression in deep-sequencing analysis (Table 2). Thus, combined microarray and deep sequencing analyses allowed us to identify a set of miRNAs that are differentially expressed in glioblastoma stem cells and normal neural stem cells.

**Table 2:** The miRNA signature of glioblastoma stem cells identified using both microarray and deep sequencing analyses.

	Microarray		Deep sequencing	
Up-regulated	Fold change	p value	Fold change	*p* value
hsa-miR-10a	93.65	<1E-07	35,949	0.00E+00
hsa-miR-10b	90.38	<1E-07	4,128	0.00E+00
hsa-miR-140-5p	12.19	<1E-07	7.2	0.00E+00
has-miR-204	9.05	<1E-07	5	0.00E+00
hsa-miR-424	8.38	<1E-07	66	0.00E+00
hsa-miR-34a	7.73	2.00E-07	2.5	4.00E-240
hsa-miR-193a-3p	6.4	8.00E-06	93	9.00E-21
hsa-miR-455-5p	5.87	1.00E-05	5.3	5.00E-214
Down-regulated				
hsa-miR-124	−13.37	8.00E-05	−10	5.00E-80
hsa-miR-874	−6.76	<1E-07	−33	1.50E-98

### Validation of the differentially expressed miRNAs using real-time RT-PCR

**Figure 2:**
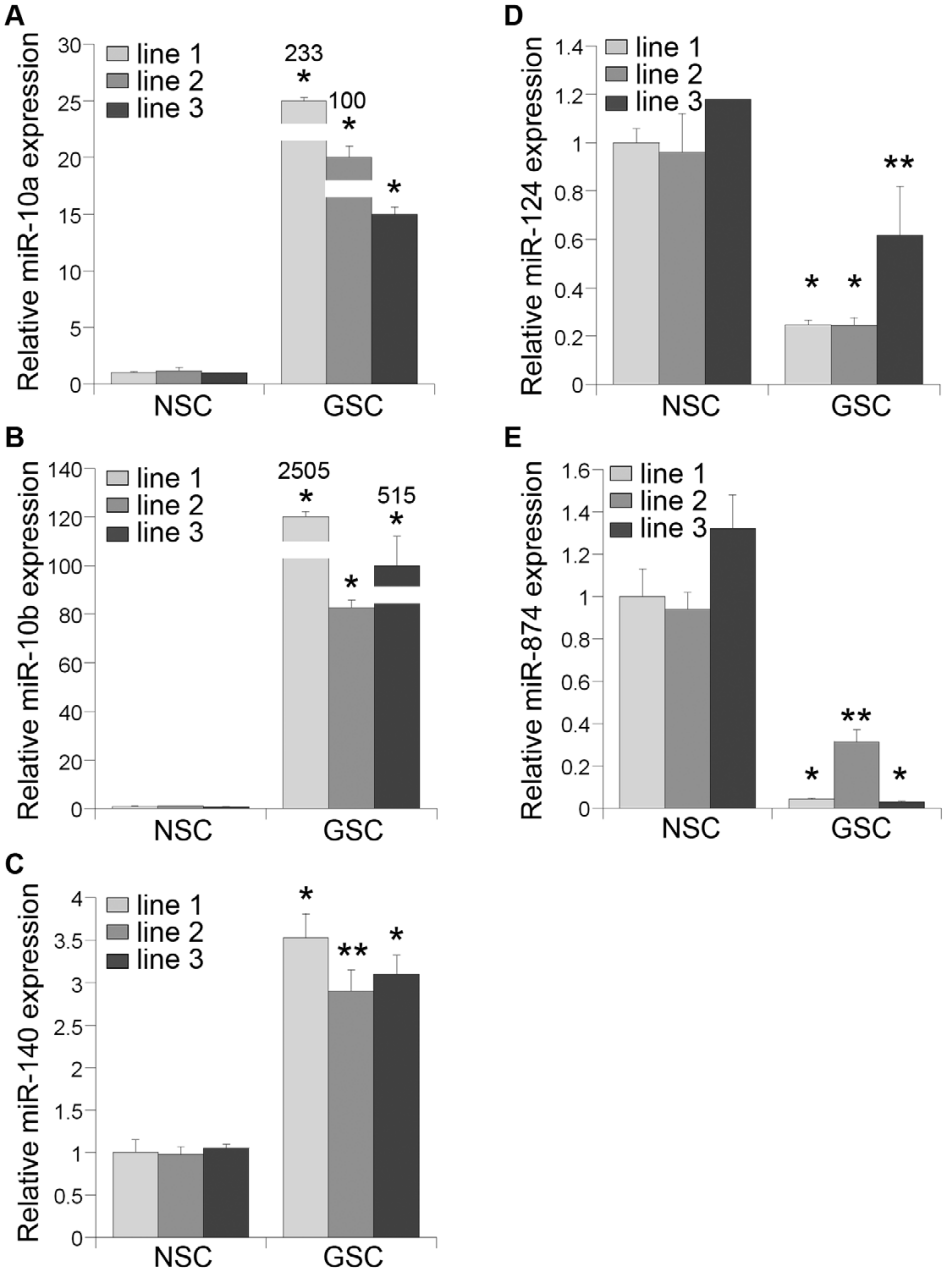
Real-time RT-PCR validation of miRNA expression in glioblastoma stem cells. The expression levels of miR-10a (**A**), miR-10b (**B**), miR-140-5p (**C**), miR-124 (**D**), and miR-874 (**E**) in three glioblastoma stem cell (GSC) lines were measured by real-time RT-PCR, and compared to their expression in three neural stem cell (NSC) lines. The expression shown in each cell line is relative to the expression in NSC1, with the expression in NSC1 as 1. Error bars are standard deviation of the mean. * p<0.001, ** p<0.005 by one way Anova test.

The distinct expression of these miRNAs in glioblastoma stem cells and neural stem cells was further validated using real-time RT-PCR analysis. RT-PCR results of the top three miRNAs that are up-regulated in glioblastoma stem cells in both microarray and deep sequencing analyses (Table 2) are shown in Fig. 2A–C. All three miRNAs showed a significant up-regulation in the three primary glioblastoma stem cell lines (GSC1–3) tested, compared to three lines of normal neural stem cells. miR-10a revealed a dramatic increase of expression in all three glioblastoma stem cell lines tested, with more than 100-fold up-regulation of expression in two of the glioblastoma stem cell lines (Fig. 2A). miR-10b exhibited even higher expression in glioblastoma stem cell lines GSC1 and GSC3, with up to 2,505-fold increase of expression in GSC1 (Fig. 2B). miR-140-5p also displayed significant increase of expression in all three glioblastoma stem cell lines tested, although with much lower fold induction (Fig. 2C).

There are two miRNAs that are down-regulated more than 5-fold in glioblastoma stem cells in both microarray and deep sequencing analyses. The expression of these two miRNAs was also validated using real-time RT-PCR assays. Both miR-124 and miR-874 exhibited reproducible decrease of expression in all three glioblastoma stem cell lines tested, compared to normal neural stem cells (Fig. 2D, E). Specifically, the relatively new miRNA miR-874 that has not been studied extensively exhibited a significant reduction of expression in all three glioblastoma stem cell lines, with more than 20-fold reduction in two of the glioblastoma stem cell lines GSC1 and GSC3 (Fig. 2E).

**Figure 3:**
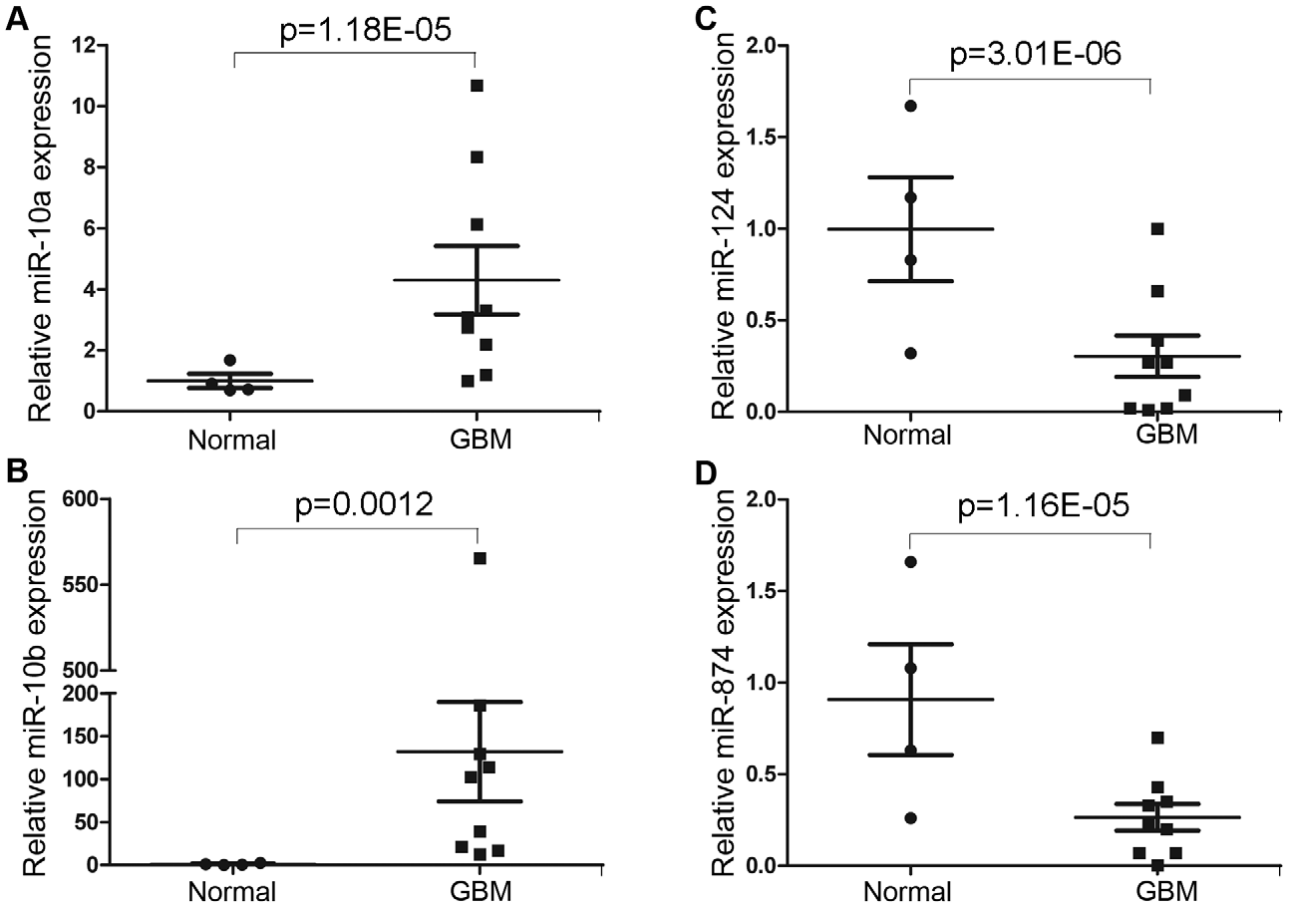
Real-time RT-PCR analysis of miRNA expression in glioblastoma tissues. The expression levels of miR-10a (**A**), miR-10b (**B**), miR-124 (**C**), and miR-874 (**D**) in 9 glioblastoma tissues and 4 normal brain tissues were determine by real-time RT-PCR analysis, shown in scatted graph and bar graph. Error bars are standard error of the mean. p value was obtained by student's *t*-test.

In addition to cultured cells, we next tested whether the miRNAs that are differentially expressed in glioblastoma stem cells and neural stem cells exhibit a distinct expression pattern in normal and glioblastoma brain tissues. For this purpose, RNAs were isolated from 9 grade IV glioblatoma multiforme brain tissue samples and 4 non-tumor normal brain tissue samples. Real-time RT-PCR analyses were performed to detect the expression of two up-regulated miRNAs and two down-regulated miRNAs. Consistent with the results from tumor stem cells, miR-10a exhibited a substantial increase of expression in most glioblastoma tissues (Fig. 3A). miR-10b also exhibited a dramatic up-regulation of expression in all of the glioblastoma tissues tested, with an average increase of 142-fold (Fig. 3B). For miRNAs that were down-regulated in glioblastoma stem cells, both miR-124 and miR-874 displayed a significant decrease of expression in most of the glioblastoma tissues tested, compared to their average expression in normal brain tissues (Fig. 3C, D).

### Target identification of the differentially-expressed miRNAs

**Figure 4:**
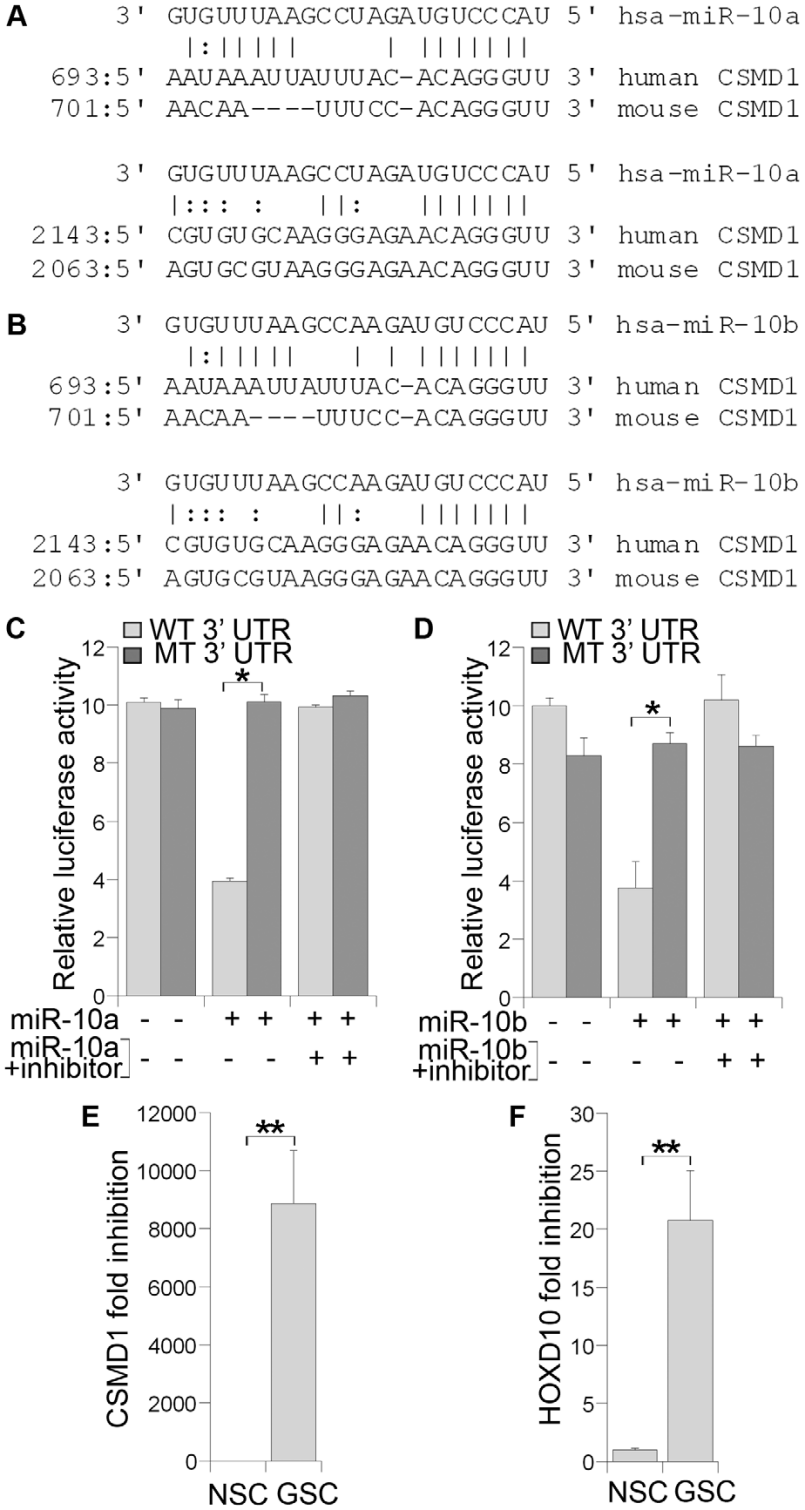
Expression of miR-10b targets in glioblastoma stem cells. **A, B.** The base-pairing of hsa-miR-10a and hsa-miR-10b with the 3′ UTR of CSMD1 gene. **C.** miR-10a-mediated repression of luciferase reporter gene downstream of 3′ UTR of CSMD1. Luciferase reporter gene under the control of wild type (WT) or mutant (MT) CSMD1 3′ UTR was transfected into HEK 293 cells along with control, miR-10a RNA duplexes, or the combination of miR-10a RNA duplexes and a miR-10a inhibitor. * p<0.001 by student's *t*-test. **D.** miR-10b-mediated repression of luciferase reporter gene downstream of 3′ UTR of CSMD1. WT or MT CSMD1 3′ UTR luciferase reporter was transfected into HEK 293 cells along with control, miR-10b RNA duplexes, or the combination of miR-10b RNA duplexes and a miR-10b inhibitor. * p<0.005 by student's *t*-test. **E.** Expression of CSMD1 in glioblastoma stem cell line 1 (GSC) and neural stem cell line 1 (NSC) determined by real-time RT-PCR analysis. **F.** Expression of HOXD10 in GSC and NSC determined by real-time RT-PCR analysis. For all panels, data shown are mean ± standard deviation of three replicates. ** p<0.01 by student's *t*-test for both panels **E** and **F**.

By using Targetscan algorithm [16], we identified CUB and SUSHI multiple domain protein 1 (CSMD1) as a candidate target for miR-10a and miR-10b, the most highly up-regulated miRNAs in glioblastoma stem cells in our profiling analyses. CSMD1 is a tumor suppressor gene that maps to chromosome 8p23, a region deleted in many tumor types [17]. Sequence analysis revealed that the seed region of both miR-10a and miR-10b could form complementary base pairs with the 3′ untranslated region (3′ UTR) of human and mouse CSMD1 mRNAs (Fig. 4A, B). To demonstrate a direct interaction between the 3′ UTR of CSMD1 and miR-10 (miR10a and miR-10b), we inserted the 3′ UTR region of human CSMD1 that contains the putative miR-10 recognition sites and flanking sequences downstream of a *Renilla* luciferase reporter gene into a siCheck vector. RNA duplexes of mature miR-10a or miR-10b were transfected into human embryonic kidney HEK293 cells along with the reporter gene. Significant repression of the reporter gene was observed in both miR-10a and miR-10b-transfected cells (Fig. 4C, D). Mutation of the miR-10 targeting sites abolished the repression (Fig. 4C, D). Furthermore, treatment of the inhibitors of miR-10a and miR-10b reversed the inhibitory effect of miR-10a and miR-10b on the luciferase reporter activity, respectively (Fig. 4C, D). These results suggest that both miR-10a and miR-10b repress CSMD1 expression through the predicted targeting sites in CSMD1 3′ UTR.

Since miR-10a and miR-10b are both up-regulated in glioblastoma stem cells relative to neural stem cells, we next examined the expression of CSMD1 in both cell types. Dramatic reduction of CSMD1 mRNA expression was detected in glioblastoma stem cells by RT-PCR analysis, compared to neural stem cells (Fig. 4E), consistent with the observation that CSMD1 expression is repressed by miR-10 (Fig. 4C, D). The homeobox transcription factor HOXD10 was identified as a tumor suppressor gene targeted by miR-10b in breast cancers recently [18]. We showed here that the HOXD10 mRNA expression is also dramatically reduced (>20-fold) in glioblastoma stem cells examined, compared to normal neural stem cells (Fig. 4F). Together, these results suggest that miR-10 targets the expression of tumor suppressor genes, CSMD1 and HOXD10, in glioblastoma stem cells.

**Figure 5:**
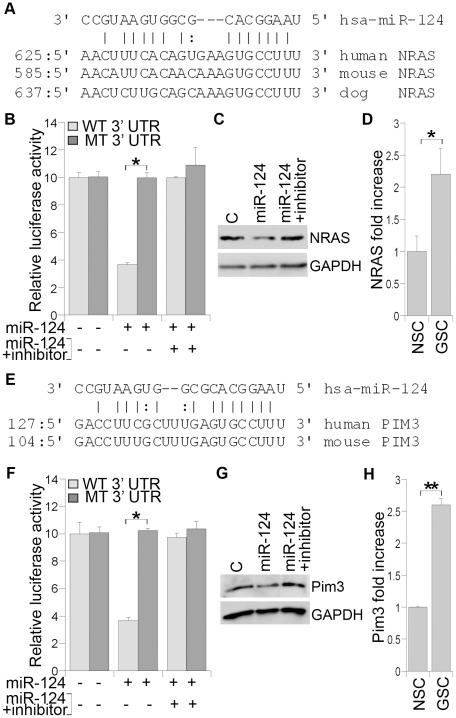
miR-124 targets NRAS and PIM3 expression. **A.** The base-pairing of hsa-miR-124 with the 3′ UTR of NRAS gene. **B.** miR-124-mediated repression of luciferase reporter gene downstream of 3′ UTR of NRAS. Luciferase reporter gene under the control of wild type (WT) or mutant (MT) NRAS 3′ UTR was transfected into HEK 293 cells along with control, miR-124 RNA duplexes, or the combination of miR-124 RNA duplexes and a miR-124 inhibitor. *p<0.001 by student's *t*-test. **C.** Western blot analysis of NRAS expression in control RNA, miR-124 RNA duplexes, or the combination of miR-124 RNA duplexes and a miR-124 inhibitor-transfected GSC1 cells. **D.** Expression of NRAS in GSC1 and NSC1 determined by real-time RT-PCR analysis. *p<0.05 by student's *t*-test. **E.** The base-pairing of hsa-miR-124 with the 3′ UTR of PIM3 gene. **F.** miR-124-mediated repression of luciferase reporter gene downstream of 3′ UTR of PIM3. Luciferase reporter gene under the control of wild type (WT) or mutant (MT) PIM3 3′ UTR was transfected into HEK 293 cells along with control, miR-124 RNA duplexes, or the combination of miR-124 RNA duplexes and a miR-124 inhibitor. *p<0.001 by student's *t*-test. **G.** Western blot analysis of PIM3 expression in control RNA, miR-124 RNA duplexes, or the combination of miR-124 RNA duplexes and a miR-124 inhibitor-transfected GSC1 cells. **H.** Expression of PIM3 in GSC1 and NSC1 determined by real-time RT-PCR analysis. **p<0.001 by student's *t*-test. For all panels, data shown are mean ± standard deviation of three replicates.

Furthermore, using the Targetscan algorithm, we predicted oncogenes NRAS and PIM3 as putative target genes of miR-124, one of the down-regulated miRNAs in glioblastoma stem cells. NRAS is a small guanine-nucleotide binding protein and one of the three RAS (KRAS, NRAS, HRAS) isoforms [19]. The RAS signaling pathway plays a crucial role in many cancers by regulating cell proliferation, differentiation, and survival [20]. Using Targetscan algorithm, miR-124 was predicted to have a targeting site at the 3′ UTR of the NRAS gene. This targeting site is conserved in human, mouse, and dog NRAS (Fig. 5A). To validate the targeting of NRAS by miR-124, we made a luciferase reporter construct with human NRAS 3′ UTR containing the predicted miR-124 targeting site and the flanking sequences inserted into the 3′UTR of a *Renilla* luciferase reporter gene in a siCHECK vector. Transfection of miR-124 RNA duplexes led to significant repression of the reporter gene (Fig. 5B). Mutation of the putative miR-124 targeting site abolished the repression (Fig. 5B). Furthermore, treatment with a miR-124 inhibitor reversed the inhibitory effect of miR-124 on the luciferase reporter activity (Fig. 5B). These results suggest that miR-124 represses NRAS expression through the predicted targeting site in NRAS 3′UTR.

Next, we tested whether miR-124 targets NRAS expression in glioblastoma stem cells. Mature miR-124 RNA duplexes were introduced into GSC1 cells using a cationic triethanolamine-core polyamidoamine (PAMAM) dendrimer-mediated small RNA delivery system [21], [22]. A control RNA duplex was included as a negative control. NRAS expression levels were examined by Western blot analysis. Reduction of NRAS protein level was detected in miR-124-transfected cells. Co-transfection of a miR-124 RNA inhibitor abolished the inhibitory effect of miR-124 on NRAS expression (Fig. 5C). This result indicates that miR-124 down-regulates endogenous NRAS expression in glioblastoma stem cells.

We also examined the expression of NRAS in glioblastoma stem cells and neural stem cells, where miR-124 exhibits differential expression. A significant increase of NRAS mRNA expression was detected in glioblastoma stem cells, compared to neural stem cells (Fig. 5D). The inverse expression pattern of NRAS and miR-124 is consistent with the observation that NRAS expression is repressed by miR-124 (Fig. 5B, C).

A putative targeting site of miR-124 was also identified in the 3′ UTR of both human and mouse PIM3, a proto-oncogene with serine/threonine kinase activity (Fig. 5E). PIM3 has been shown to promote tumor cell growth through modulating cell cycle regulators [23], [24]. To validate the targeting of PIM3 by miR-124, we made a luciferase reporter construct with human PIM3 3′ UTR containing the predicted miR-124 targeting site and the flanking sequences inserted into the 3′UTR of a *Renilla* luciferase reporter gene. Transfection of miR-124 led to significant repression of the reporter gene and mutation of the putative miR-124 targeting site abolished the repression (Fig. 5F). Furthermore, treatment with a miR-124 inhibitor reversed the inhibitory effect of miR-124 on the luciferase reporter activity (Fig. 5F). These results suggest that miR-124 represses PIM3 expression through the predicted targeting site in its 3′ UTR.

To test whether miR-124 targets PIM3 expression in glioblastoma stem cells, mature miR-124 RNA duplexes were introduced into GSC1 cells using the dendrimer-mediated delivery system [21], [22]. A control RNA duplex was included as a negative control. Reduction of PIM3 protein level was detected in miR-124-transfected cells by Western blot analysis. Co-transfection of a miR-124 RNA inhibitor abolished the inhibitory effect of miR-124 on PIM3 expression (Fig. 5G). This result indicates that miR-124 down-regulates endogenous PIM3 expression in glioblastoma stem cells. Moreover, a significant increase of PIM3 mRNA expression was detected in the glioblastoma stem cells tested, compared to neural stem cells (Fig. 5H), further supporting the idea that miR-124 represses PIM3 expression.

**Figure 6:**
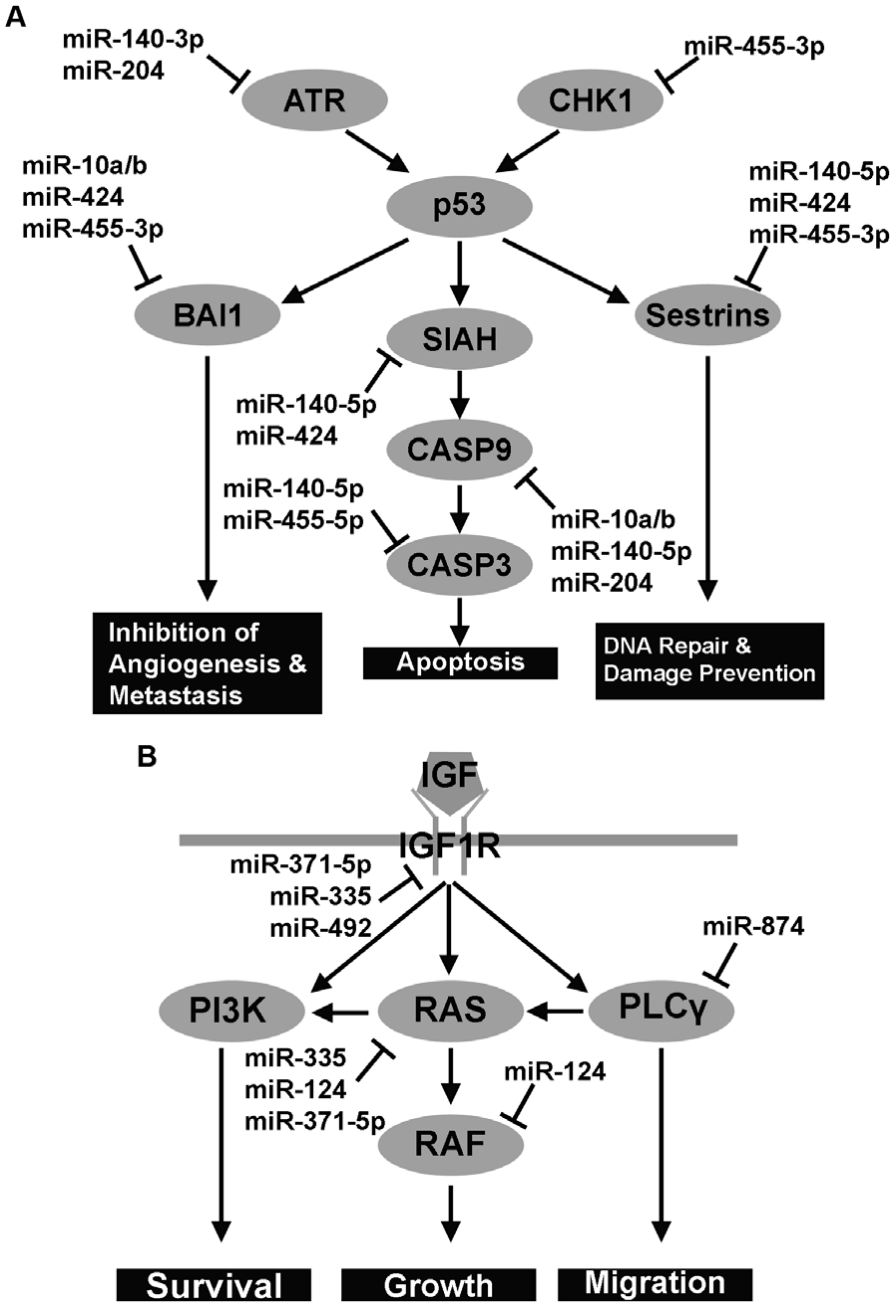
Pathways targeted by deregulated miRNAs in glioblastoma stem cells. Common miRNA targets were subjected to DAVID functional annotation with KEGG pathway analysis. **A.** The up-regulated miRNAs in glioblastoma stem cells were predicted to target the p53 pathway. The p53-centered pathway has been shown to regulate cell cycle, apoptosis, angiogenesis, metastasis, and genome stability. **B.** The down-regulated miRNAs were predicted to target components of the IGF pathway. Various components of the IGF signaling pathways were targeted by down-regulated miRNAs. The IGF pathway has been shown to enhance cell growth, survival, and migration.

It is increasingly clear that miRNAs are important regulators of key signaling pathways implicated in tumorigenesis. Using Kyoto Encyclopedia of Genes and Genomes (KEGG) pathway analysis, we compared the predicted targets of miRNAs that showed more than 5-fold up-regulation or down-regulation in glioblastoma stem cells in our microarray analysis. Seven of the ten miRNAs that were up-regulated more than 5-fold in glioblastoma stem cells are predicted to have components of the p53 pathway as common targets (Fig. 6A). The p53 pathway has been shown to be involved in cell cycle arrest, apoptosis, inhibition of cell migration, inhibition of angiogenesis, and affect genomic stability [25]. In contrast, five out of eight miRNAs that exhibited more than 5-fold down-regulation in glioblastoma stem cells were predicted to target components of the IGF pathway (Fig. 6B) that has been implicated in promoting cell growth, survival and migration [26].

## Discussion

In the present study, we investigated genome-wide miRNA expression in tumor stem cell populations of glioblastoma, the most frequent and malignant primary brain tumor. In spite of recent improvement of surgical and radiotherapeutic techniques, the prognosis for glioblastoma patients is still very poor. The search for molecular targets is fundamental to develop effective treatments for glioblastoma.

Global profiling is one of the most effective approaches to identify abnormally expressed miRNAs in tumor genomes. In this study, we used three different technical platforms to determine the differential expression of miRNAs in glioblastoma stem cells and neural stem cells. We combined the microarray platform with the newly emerged small RNA deep sequencing technology to profile miRNA expression in glioblastoma stem cells and normal neural stem cells and validated the profiling results using quantitative RT-PCR. Although the absolute fold change obtained from each platform is different due to the different sensitivity of the techniques, the trend of the change for the miRNAs studied is consistent. The miRNA expression profile could clearly distinguish glioblastoma stem cells from normal neural stem cells, allowing us to identify a miRNA signature of glioblastoma stem cells that were significantly up-regulated or down-regulated in glioblastoma stem cells, relative to neural stem cells.

In line with our findings that a set of miRNAs are differentially expressed in glioblastoma stem cells and normal neural stem cells, certain miRNAs also exhibit distinct expression profiles in glioblastoma tissues and normal brain tissues. For example, we demonstrate for the first time in this study that the expression of miR-874 is dramatically reduced in glioblastoma tissues, compared to normal brain tissues. miR-124, another miRNA that was down-regulated in glioblastoma stem cells, also exhibited reduced expression in glioblastoma tissues in this study, consistent with the results of previous glioblastoma tumor tissue profiling [12], [14], [27]–[31].

In this study, we show that miR-10b is highly expressed in both glioblastoma stem cells and in glioblastoma tumor tissues. Up-regulation of miR-10b was also observed in other glioblastoma samples [12], [27], [28], suggesting an important role for miR-10b in glioblastoma tumorigenesis. Moreover, a recent study revealed that miR-10b expression is inversely correlated with glioblastoma patient survival [32]. Interestingly, miR-10b was also found to be up-regulated in breast cancer, leukemia, and pancreatic cancer and promote tumor invasion and metastasis in breast cancer [18], [33], [34]. Together, these results suggest that some miRNAs, such as miR-10b, may function as a global oncogene to stimulate tumorigenesis in multiple tissues. Likewise, miR-124 is also frequently down-regulated in other cancers, such as medulloblastoma, hepatocellular carcinoma, and oral squamous carcinoma [35]–[37], suggesting that it may function as a general tumor suppressor. Therefore the knowledge of miRNAs that we have obtained for glioblastoma stem cells may shed lights to other cancer stem cells.

Pathway analysis revealed that most of the significantly up-regulated miRNAs, with more than 5-fold increase in glioblastoma stem cells as shown in our microarray analysis, have putative targets in a common pathway, the p53 pathway. The dysregulation of the p53 pathway has been shown to be an underlying mechanism for tumorigenesis [25], thus the up-regulated miRNAs may function as oncomiRs by targeting the p53 pathway if their role in regulating the p53 pathway is confirmed. On the other hand, most of the down-regulated miRNAs, with more than 5-fold decrease in glioblastoma stem cells, share their predicted targets in the IGF signaling. Repression of the IGF signaling has been shown to inhibit tumorigenesis [26]. Thus, these down-regulated miRNAs may assume a role of tumor suppressors by targeting components of the IGF pathway if their role in regulating the IGF signaling is confirmed. The identification of miRNAs as oncogenes or tumor suppressors holds the promise of identifying novel diagnostic markers or molecular targets for antitumor therapies. The prediction that the differentially expressed miRNAs have the ability to target multiple components in one or more pathways makes them potential molecular targets for cancer therapy.

Glioblastoma stem cells represent a subpopulation of cancer cells with extraordinary capacities to promote tumor growth, invasion and therapeutic resistance, making them an ideal target cell population for anti-glioblastoma therapies. However, a major challenge is to define functional and molecular features that can distinguish cancer stem cells from normal stem cells in order to develop therapeutic strategies that specifically target the tumor population, but leave normal stem cells intact. Therefore comparing miRNA expression between glioblastoma stem cells and normal neural stem cells is highly relevant in that it may lead to the identification of glioblastoma stem cell-specific miRNAs, thus resulting in the development of novel glioblastoma therapies by targeting only tumor stem cells. We performed the comparison of miRNA expression between glioblastoma stem cells from adult glioblastoma patients and normal neural stem cells from human fetal brains instead of human adult brains due to the inaccessibility of normal human adult brain tissues containing neural stem cells. Although differences do exist between embryonic neural stem cells and adult neural stem cells, it has been proposed that embryonic neural stem cells resemble adult neural stem cells in many ways [38]. Therefore this comparison will provide useful information regarding glioblastoma stem cell-specific miRNA expression and provide a basis for strategic targeting glioblastoma stem cells through modulation of tumor stem cell-specific miRNA expression.

miRNAs have been shown to be involved in tumor initiation and progression, functioning as oncogenes or tumor suppressor [9], [10]. Therefore, modulation of miRNA expression provides great hope for potential cancer therapy [39]. Furthermore, since each miRNA may have more than one targets, miRNA-based gene therapy offers the therapeutic appeal of targeting multiple gene networks that are controlled by a single miRNA [9]. Strategies for miRNA-based cancer therapy include overexpression of tumor suppressor miRNAs and targeting oncogenic miRNAs using their antagonists. Based on the miRNA signature that we have identified in glioblastoma stem cells, we may be able to develop targeted glioblastoma therapies by inhibiting the up-regulated miR-10a or miR-10b function using miR-10 antogonists or overexpressing the down-regulated miR-124 or miR-874. Of note,16 miRNAs that are up-regulated in glioblastoma stem cells (>1.5-fold, Table S1) in our study, including miR-10a and miR-10b, are also up-regulated in malignant astrocytomas (glioblastomas and anaplastic astrocytomas) in a genome-wide miRNA expression profiling between malignant astrocytomas and normal brain samples [40]. Eleven miRNAs that are down-regulated in glioblastoma stem cells (>1.5-fold, Table S1) in our study, including miR-124, are also down-regulated in malignant astrocytomas.

Moreover, the prognosis of glioblastoma patients remains poor. Biomarkers for this disease are needed for early detection of tumor progression [41]. The miRNA signature that we identified here may be used as biomarkers to differentiate glioblastoma stem cells from normal neural stem cells. Recently, a miRNA signature was identified in the peripheral blood of glioblastoma patients [41]. Interestingly, several of the miRNAs that showed elevated expression in the blood samples of glioblastoma patients (vs healthy control) also exhibited increased expression in glioblastoma stem cells (vs normal neural stem cells) in our study (Table S1), including miR-424, miR-148a, miR-362-3p, miR-30d, miR-128. These miRNAs may therefore represent easily accessible biomarkers that can be used for diagnostic purposes in glioblastoma patients.

## Materials and Methods

### Ethics statement

The derivation of PBT003 (GSC1) and PBT017 (GSC2) has been described by Brown et al [42]. PBT707 (GSC3) is a de novo cell line derived from anonymized leftover tissues with the approval of the City of Hope Institutional Review Board. The study involves the use of completely anonymized specimens. No informed consent is involved. NOD-scid IL2Rgamma^null^ (NSG) mice (6–8weeks) were used for glioblastoma stem cell transplantation. Tumor cell transplantation was performed under the IACUC protocol 05050 approved by the City of Hope Institutional Animal Care and Use Committee.

### Glioblastoma stem cell and neural stem cell culture

Glioblastoma stem cells were derived from newly diagnosed WHO grade IV glioblastoma tissues. Specifically, freshly isolated glioblastoma tissues were minced with sterile scissors and dissociated into single cells using 400 units/ml of collagenase III in DMEM/F12 medium supplemented with 5 µg/ml heparin, 1× B27 (GIBCO/BRL), and 2 mM L-glutamine. Dissociated cells were then centrifuged at 1,200 rpm for 5 min and the supernatant was discarded. To eliminate red blood cells, the resultant cells were incubated in 10 ml red blood cell lysis buffer (Invitrogen) for 10 min. Cells were centrifuged again at 1,200 rpm for 5 min and supernatant was discarded. The resultant cells were resuspended in DMEM/F12 medium supplemented with 20 ng/ml EGF, 20 ng/ml FGF, 5 µg/ml heparin, 1× B27 (GIBCO/BRL), and 2 mM L-glutamine and cultured in this medium thereafter. Tumor spheres appeared around one week in culture. Normal human neural stem cells were derived from primary human brain tissues and maintained in the same culture media. Specifically, human fetal brain tissues (Biosciences Resources) were dissociated in cold Hanks balance salt solution (HBSS) using polished glass pipette. The resultant cells were centrifuged and resuspended in DMEM/F12 medium supplemented with 0.5× B27, 25 µg/ml insulin, 20 µg/ml apo-transferrin, 30 nM sodium selenite, 20 nM progesterone, 100 mM putrescine, 20 ng/ml FGF and 10 ng/ml LIF. The initial culture was split at 1∶2 each day for 4 days, followed by media change every other day till day 21. Human neurospheres started to appear around day 14. The spheres were split around day 21 with Accutase (Sigma) and cultured in DMEM/F12 medium supplemented with 20 ng/ml EGF, 20 ng/ml FGF, 5 µg/ml heparin, 1× B27 (GIBCO/BRL), and 2 mM L-glutamine thereafter. Both tumor spheres and normal neurospheres were characterized for their self-renewal and multipotency. Glioblastoma stem cell spheres were also characterized for their ability to derive brain tumors.

For differentiation, both glioblastoma stem cells and neural stem cells were induced into differentiation using 0.5% fetal bovine serum and 1 µM all-trans retinoic acid. For *in vivo* tumor formation assays, 2×10^5^ dissociated glioblastoma stem cells were injected into cerebral cortex of NSG mice by stereotaxic injection. The coordinates for the injection were AP 0.6 mm, ML +1.6 mm and DV −2.6 mm. Brains were harvested 5 weeks after cell transplantation. Frozen brains were cut into 20 µm coronal sections, followed by Hematoxylin & Eosin (H&E) staining.

### Glioblastoma stem cell transfection using a dendrimer-based delivery system

Spheres of glioblastoma stem cells were dissociated and seeded into 24-well plates at 2×10^5^ cells per well in 300 µl of medium. The generation-5 (G5) dendrimers and Opti-MEM solution were mixed by vortex for 10 seconds, and incubated at room temperature (RT) for 10 min. The miRNA duplexes or the combination of miRNAs and their short hairpin RNA inhibitors were added into dendrimer/Opti-MEM solution in a total volume of 100 µl, mixed gently for 10 sec, and incubated at RT for 25 min. The nitrogen-to-phosphorus (N/P) ratio of the dendrimer/RNA complex is 5. The 100 µl dendrimer/RNA complex was added into 300 µl cell suspension in each well of 24-well plates, shake gently and put back to CO_2_ incubator. Forty-eight hr after transfection, cells were collected and subjected to Western blot analysis.

### Western blot analysis

Whole cell extracts of glioblastoma stem cells were prepared using RIPA buffer (50 mM Tris-HCl pH 7.5, 150 mM NaCl, 1% NP40, 0.5% deoxycholate and 0.1% SDS) containing protease inhibitor cocktail (Roche). Western blotting was performed with anti-NRAS (sc-31, 1∶100) and anti-PIM3 (sc-98959, 1∶100) antibodies from Santa Cruz.

### Glioblastoma stem cell transplantation

NSG mice (6–8weeks) were used for glioblastoma stem cell transplantation. Tumor cell transplantation was performed under the IACUC protocol 05050 approved by the City of Hope Institutional Animal Care and Use Committee. 5×10^4^ dissociated glioblastoma stem cells were injected into the front lobe of forebrains by stereotaxic injection. The coordinates for the injection were AP 0.6 mm, ML +1.6 mm and DV −2.6 mm.

### Reporter construct preparation

DNA fragments encoding the 3′ UTR of putative miRNA targets were cloned into psiCHECK 2 (Promega), downstream of a *Renilla* luciferase reporter gene. The PCR primers that were used for 3′ UTR cloning of each gene are as follows: CSMD1 forward: 5′ GAT CCT CGA GCT GTT CTG TCG CAG AAT G 3′ and CSMD1 reverse: 5′ GAT CGC GGC CGC GTC AGC ATT TTG CAC CTA G3′; PIM3 forward: 5′ GAT CCT CGA GGC TTG TGA GGA GCT GCA C 3′ and PIM3 reverse: 5′ GAT CGC GGC CGC GGA AAC TTG TCA GGT CAC C 3′; NRAS forward: 5′ GAT CCT CGA GCT GGA GGA GAA GTA TTC CTG 3′ and NRAS reverse: 5′ GAT CGC GGC CGC TGC AAA TGT AGA GCT TTC TGG 3′. Corresponding miRNA binding sites on the 3′ UTRs were mutated by site-directed mutagenesis according to the manufacturer's instructions (Stratagene). The binding site of hsa-miR-124 on the 3′ UTR of PIM3 and NRAS was mutated from GTGCCTT to GTGGACA ; the binding site of hsa-miR-10b on the 3′ UTR of CSMD1 was mutated from ACAGGGT to ACAGTCC .

### Transfection and reporter assay

We transfected plasmid DNA or DNA-miRNA mixture into HEK293 cells using Transfectin (Bio-Rad) as described [43]–[45]. miR-10a, miR-10b or miR-124 RNA duplexes and/ or their correspondent RNA inhibitors (Dharmacon) were mixed in 50 µl serum free media with Transfectin, incubated at RT for 20 min. Negative controls for miRNA and their hairpin inhibitors were included. The final concentration of miRNAs or their inhibitors was 20 nM. The resultant mixture was added dropwise to HEK293 cells in a 24-well plate with 450 µl medium per well to a total volume of 500 µl per well. The transfected cells were harvested 48 h after transfection and subjected to subsequent reporter assays as described [46]. Reporter *Renilla* luciferase activity was measured 48 hrs after transfection using Dual Luciferase Assay kit (Promega). The *Renilla* luciferase activity was normalized by firefly luciferase internal control and expressed as relative luciferase activity. The miR-10a RNA duplex sense sequence is 5′ TAC CCT GTA GAT CCG AAT TTG TG 3′. The miR-10b RNA duplex sense sequence is 5′ TAC CCT GTA GAA CCG AAT TTG TG 3′. The miR-124 RNA duplex sense sequence is 5′ TAA GGC ACG CGG TGA ATG CC 3′. And the control RNA duplex sense sequence is 5′ UCA CAA CCU CCU AGA AAG AGU AGA 3′.

### Real-time RT-PCR analysis

For miRNA expression, total RNAs were reversely transcribed and quantified by real-time RT-PCR with TaqMan MicroRNA Assay kit (Applied Biosystems). The expression of specific miRNAs was normalized using human U18 snRNA. For mRNA expression, putative miRNA targets were quantified by iTaq SYBR Green Supermix with ROX (Bio-Rad). Primers used for RT-PCR include PIM3 forward: 5′ AGC TCA AGC TCA TCG ACT TC 3′ and PIM3 reverse: 5′ TAG CGG TGG TAG CGG ATC 3′; NRAS forward: 5′ CCA TGA GAG ACC AAT ACA TGA G 3′ and NRAS reverse: 5′ GCT TAA TCT GCT CCC TGT AG 3′; HOXD10 forward: 5′ TTC CCG AAG AGA GGA GCT G 3′ and HOXD10 reverse: 5′ CTG CCA CTC TTT GCA GTG AG 3′; CSMD1 forward: 5′ GCA GAA ATG CTT ACT GAG GAT G 3′ and CSMD1 reverse: 5′ AGA ACC CTC AAA CTG CAA CTG 3′; GAPDH forward: 5′ ATC ACC ATC TTC CAG GAG C 3′ and GAPDH reverse 5′ CCT TCT CCA TGG TGG TGA AG 3′.

### miRNA microarray and deep sequencing analysis

Total RNAs were extracted from glioblastoma stem cells or human neural stem cells by TRIzol (Invitrogen) method according to manufacturer's protocol. Ten µg of RNA was used for miRNA microarray using Exiqon platform. One µg of RNA was used for deep sequencing using Illumina Genome Analyser II (GAII). All data are MIAME compliant.

### Pathway analysis

Common putative targets of either the down-regulated or the up-regulated miRNAs were uploaded onto the Database for Annotation, Visualization and Integrated Discovery (DAVID) Functional Annotation Bioinformatics Microarray Analysis (http://david.abcc.ncifcrf.gov/). Kyoto Encyclopedia of Genes and Genomes (KEGG) pathway in DAVID was used to depict the biological meanings of the common miRNA targets.

## Supporting Information

Table S1 **Up-regulated and down-regulated miRNAs (>1.5 fold) in human glioblastoma stem cells, compared to human neural stem cells.**
(DOC)
