# Supplementary material for: Genome-Wide Profiling Identified a Set of miRNAs that Are Differentially Expressed in Glioblastoma Stem Cells and Normal Neural Stem Cells
Source: PLoS One. 2012 Apr 30;7(4):e36248. doi: 10.1371/journal.pone.0036248 (PMC3340364; doi:10.1371/journal.pone.0036248)
Supplement: Table S1 — Up-regulated and down-regulated miRNAs (>1.5 fold) in human glioblastoma stem cells, compared to human neural stem cells. (DOC) [file pone.0036248.s001.doc]

**Table S1. Up-regualted and down-regulated miRNAs (>1.5 fold) in human glioblastoma stem cells, compared to human neural stem cells.**

| miRNA | Chromosomal  location | Fold-Change | *p*-value |
| --- | --- | --- | --- |
| **Up-regulated** |  |  |  |
| hsa-miR-10a | 17q21.32 | 93.64622 | 3.28E-10 |
| hsa-miR-10b | 2q31.1 | 90.38293 | 2.06E-09 |
| hsa-miR-140-3p | 16q22.1 | 14.10262 | 1.62E-10 |
| hsa-miR-140-5p | 16q22.1 | 12.19325 | 5.56E-09 |
| hsa-miR-204 | 9q21.12 | 9.05353 | 4.08E-08 |
| hsa-miR-424 | Xq26.3 | 8.38382 | 2.53E-08 |
| hsa-miR-34a | 1p36.22 | 7.73283 | 2.21E-07 |
| hsa-miR-193a-3p | 17q11.2 | 6.39914 | 7.95E-06 |
| hsa-miR-455-5p | 9q32 | 5.87119 | 1.48E-05 |
| hsa-miR-455-3p | 9q32 | 5.40680 | 9.32E-05 |
| hsa-miR-9* | 1q22 | 4.43204 | 8.28E-08 |
| hsa-miR-10a* | 17q21.32 | 3.89600 | 1.92E-05Principio del formulario  Final del formulario |
| hsa-miR-148a | 7p15.2 | 3.16202 | 1.93E-05 |
| hsa-miR-488 | 1q25.2 | 2.77759 | 1.69E-03 |
| hsa-miR-196a^1^ | 17q21.32 | 2.76521 | 2.69E-03 |
| hsa-miR-182 | 7q32.2 | 2.75689 | 1.52E-03 |
| hsa-miR-96 | 7q32.2 | 2.61400 | 1.61E-03 |
| hsa-miR-193b | 16p13.12 | 2.57981 | 4.90E-06 |
| hsa-miR-27a | 19p13.13 | 2.53543 | 1.73E-07 |
| hsa-miR-196b | 7p15.2 | 2.50868 | 3.54E-03 |
| hsa-miR-10b* | 2q31.1 | 2.40614 | 8.55E-03 |
| hsa-miR-29b^2^ | 7q32.3 | 2.39299 | 3.27E-07 |
| hsa-miR-23a | 19p13.13 | 2.34219 | 9.88E-08 |
| hsa-miR-107 | 10q23.31 | 2.31374 | 4.42E-07 |
| hsa-miR-542-3p | Xq26.3 | 2.28873 | 4.59E-03 |
| hsa-miR-9^3^ | 1q22 | 2.24593 | 1.27E-07 |
| hsa-miR-365a^4^ | 16p13.12 | 2.20050 | 2.54E-06 |
| hsa-miR-450a | Xq26.3 | 2.11076 | 1.43E-02 |
| hsa-miR-100 | 11q24.1 | 2.04624 | 5.71E-07 |
| hsa-miR-105 | Xq28 | 1.99529 | 3.86E-03 |
| hsa-miR-363 | Xq26.2 | 1.99346 | 1.23E-02 |
| hsa-miR-105* | 4q24 | 1.88156 | 1.57E-02 |
| hsa-miR-106b | 7q22.1 | 1.86896 | 5.63E-06 |
| hsa-miR-15b | 3q25.33 | 1.79677 | 3.00E-06 |
| hsa-miR-21 | 17q23.1 | 1.76837 | 3.03E-05 |
| hsa-miR-376c | 14q32.31 | 1.76028 | 5.72E-04 |
| hsa-miR-93 | 7q22.1 | 1.74398 | 6.15E-06 |
| hsa-miR-99b | 19q13.41 | 1.73839 | 1.77E-05 |
| hsa-miR-155 | 21q21.3 | 1.72212 | 1.59E-02 |
| hsa-miR-33a | 22q13.2 | 1.72056 | 8.82E-05 |
| hsa-miR-876-3p | 9p21.1 | 1.68669 | 4.45E-02 |
| hsa-miR-362-3p | Xp11.23 | 1.67653 | 4.12E-02 |
| hsa-miR-25 | 7q22.1 | 1.66555 | 1.31E-04 |
| hsa-let-7i | 12q14.1 | 1.66413 | 1.12E-05 |
| hsa-miR-423-3p | 17q11.2 | 1.64838 | 2.50E-04 |
| hsa-miR-34b | 11q23.1 | 1.62786 | 9.72E-05 |
| hsa-miR-16-2* | 3q25.33 | 1.62586 | 1.81E-03 |
| hsa-miR-29a | 7q32.3 | 1.61583 | 9.27E-06 |
| hsa-miR-30d | 8q24.2 | 1.61189 | 9.38E-04 |
| hsa-miR-320 | 8p21.3 | 1.60864 | 8.71E-05 |
| hsa-miR-181c | 19p13.13 | 1.56262 | 1.30E-02 |
| hsa-miR-128a | 2q21.3 | 1.55521 | 4.20E-02 |
| hsa-miR-21* | 17q23.1 | 1.54998 | 2.94E-02 |
| hsa-let-7d | 9q22.32 | 1.53430 | 1.10E-03 |
| hsa-miR-450b-5p | Xq26.3 | 1.53397 | 3.49E-02 |
| **Down-regulated** |  |  |  |
| hsa-miR-371-5p | 19q13.42 | -15.26715 | 8.52E-11 |
| hsa-miR-124^5^ | 8p23.1 | -13.37236 | 8.33E-05 |
| hsa-miR-335 | 7q32.2 | -13.23553 | 1.43E-09 |
| hsa-miR-492 | 12q22 | -7.76626 | 5.38E-08 |
| hsa-miR-874 | 5q31.2 | -6.76244 | 1.53E-08 |
| hsa-miR-30b* | 8q24.22 | -6.54116 | 2.73E-09 |
| hsa-miR-193a-5p | 17q11.2 | -5.76493 | 2.38E-08 |
| hsa-miR-602 | 9q34.3 | -5.74746 | 2.63E-08 |
| hsa-miR-346 | 10q23.2 | -5.69712 | 4.97E-02 |
| hsa-miR-663 | 20p11.1 | -5.37200 | 1.31E-02 |
| hsa-miR-25* | 7q22.1 | -4.97894 | 2.47E-06 |
| hsa-miR-219-5p^6^ | 6p21.32 | -4.91797 | 5.92E-07 |
| hsa-miR-184 | 15q25.1 | -4.87568 | 4.42E-08 |
| hsa-miR-135a^7^ | 3p21.1 | -4.87064 | 1.94E-07 |
| hsa-miR-584 | 5q32 | -4.60287 | 1.77E-08 |
| hsa-miR-665 | 14q32.2 | -4.35369 | 7.57E-09 |
| hsa-miR-638 | 19p13.2 | -3.47384 | 3.88E-04 |
| hsa-miR-503 | Xq26.3 | -3.43502 | 1.20E-08 |
| hsa-miR-628-3p | 15q21.3 | -3.42905 | 1.71E-07 |
| hsa-miR-381 | 14q32.31 | -3.33814 | 2.55E-07 |
| hsa-miR-7^8^ | 9q21.32 | -2.91031 | 4.96E-04 |
| hsa-miR-92b | 1q22 | -2.90543 | 2.73E-07 |
| hsa-miR-149* | 2q37.3 | -2.87318 | 1.44E-03 |
| hsa-miR-135b | 1q32.1 | -2.87250 | 4.47E-07 |
| hsa-miR-302d* | 4q25 | -2.77368 | 2.82E-03 |
| hsa-miR-498 | 19q13.42 | -2.75015 | 2.70E-03 |
| hsa-miR-766 | Xq24 | -2.49979 | 1.89E-03 |
| hsa-miR-138^9^ | 3p21.32 | -2.48259 | 9.79E-07 |
| hsa-miR-623 | 13q32.3 | -2.43864 | 3.92E-03 |
| hsa-miR-519c-5p | 19q13.42 | -2.38133 | 1.61E-05 |
| hsa-miR-182* | 7q32.2 | -2.26786 | 4.18E-02 |
| hsa-miR-494 | 14q32.31 | -2.15454 | 3.51E-06 |
| hsa-miR-129-5p^10^ | 7q32.1 | -2.13428 | 6.47E-04 |
| hsa-miR-513-5p | 11q23.1 | -2.12005 | 9.55E-03 |
| hsa-miR-200b* | 1p36.33 | -2.04703 | 5.69E-05 |
| hsa-miR-634 | 17q24.2 | -2.02966 | 6.14E-06 |
| hsa-miR-654-5p | 14q32.31 | -2.01663 | 7.48E-04 |
| hsa-miR-518b | 19q13.42 | -1.98208 | 1.05E-03 |
| hsa-miR-658 | 22q13.1 | -1.94699 | 1.80E-06 |
| hsa-miR-373* | 19q13.42 | -1.91189 | 4.25E-03 |
| hsa-miR-30c-2* | 6q13 | -1.88988 | 2.12E-06 |
| hsa-miR-130a | 11q12.1 | -1.87039 | 3.78E-05 |
| hsa-miR-557 | 1q24.2 | -1.83994 | 1.62E-03 |
| hsa-miR-551a | 1p36.32 | -1.82115 | 2.53E-03 |
| hsa-miR-637 | 19p13.3 | -1.81083 | 2.07E-02 |
| hsa-miR-518c* | 19q13.42 | -1.77801 | 4.15E-05 |
| hsa-miR-525-5p | 19q13.42 | -1.75943 | 4.76E-02 |
| hsa-miR-596 | 8p23.3 | -1.74891 | 1.75E-03 |
| hsa-miR-552 | 1p34.3 | -1.72726 | 7.31E-04 |
| hsa-miR-625* | 14q23.3 | -1.71695 | 7.00E-04 |
| hsa-miR-183* | 7q32.2 | -1.70781 | 1.41E-03 |
| hsa-miR-187* | 18q12.2 | -1.70468 | 1.12E-02 |
| hsa-miR-544 | 14 | -1.69268 | 4.74E-02 |
| hsa-miR-891a | Xq27.3 | -1.67598 | 1.29E-02 |
| hsa-miR-519e* | 19q13.42 | -1.67235 | 1.56E-02 |
| hsa-miR-933 | 2q31.1 | -1.66767 | 5.19E-05 |
| hsa-miR-939 | 8q24.3 | -1.66214 | 5.40E-03 |
| hsa-miR-214 | 1q24.3 | -1.64500 | 6.92E-03 |
| hsa-miR-671-5p | 7q36.1 | -1.64192 | 7.94E-05 |
| hsa-miR-137 | 1p21.3 | -1.63014 | 3.96E-02 |
| hsa-miR-92b* | 1q22 | -1.54966 | 4.57E-02 |
| hsa-miR-525-3p | 19q13.42 | -1.54729 | 1.45E-02 |
| hsa-miR-19a | 13q31.3 | -1.51239 | 1.90E-04 |
| hsa-miR-409-5p | 14q32.31 | -1.51024 | 5.12E-03 |
|  |  |  |  |

^1-10^For miRNAs that have more than one primary precursors, the chromosomal location of the first primary precursor is shown in the table. The chromosomal locations of other primary miRNAs include: 1) hsa-miR-196a-2: 12q13.13; 2) hsa-miR-29b-2: 1q32.2; 3) hsa-miR-9-2: 5q14.3, hsa-miR-9-3: 15q26.1; 4) hsa-miR-365b: 17q11.2; 5) hsa-miR-124-2: 8q12.3, hsa-miR-124-3: 20q13.33; 6) hsa-miR-219-2-5p: 9q34.11; 7) hsa-miR-135a-2: 12q23.1; 8) hsa-miR-7-2: 15q26.1, hsa-miR-7-3: 19p13.3; 9) hsa-miR-138-2: 16q13; and 10) hsa-miR-129-2-5p: 11p11.2.
